# The complete mitochondrial genome of *Pidorus atratus* (Lepidoptera: Zygaenoidea: Zygaenidae)

**DOI:** 10.1080/23802359.2018.1450670

**Published:** 2018-04-10

**Authors:** Zihui Wang, Shun Yao, Xiaoyue Zhu, Jiasheng Hao

**Affiliations:** Laboratory of Molecular Evolution and Biodiversity, College of Life Sciences, Anhui Normal University, Wuhu, PR China

**Keywords:** Mitochondrial genome, Zygaenoidea, Zygaenidae, *Pidorus atratus*

## Abstract

The complete mitochondrial genome (mitogenome) of *Pidorus atratus* (Lepidoptera: Zygaenoidea: Zygaenidae) is described in this study. The circular molecule is 15,383 bp in length and contains 37 typical mitochondrial genes and one non-coding AT-rich region. All protein-coding genes (PCGs) start with ATN, except for cox1 gene with CGA; 10 of the 13 PCGs harbour the typical stop codon TAA, whereas cox1, cox2, and nad4 end with a single T. Two ribosomal RNA genes (rRNAs) are 1366 bp and 544 bp in length, respectively. The AT-rich region is 658 bp in size and harbours several features characteristic of the lepidopterans, including the motif ATAGA followed by a 20 bp poly-T stretch and a microsatellite-like (TA)_9_ element. The complete mitogenome data would be useful for further understanding the taxonomy and phylogeny of Zygaenoidea.

Zygaenoidea is one of the superfamilies of lepidopterans, consisting of about 12 families, including the Zygaenidae and Limacodidae (van Nieukerken et al. 2011). Due to their unique biological characteristics, Zygaenoidea is of great interest to lepidopterists and evolutionary biologists, with its internal phylogeny and phylogenetic relationships with its closely related lepidopteran groups still standing as a controversial issue (Liu et al. 2016). As far as we knew, up to the present, more than 200 complete or near-complete lepidopteran mitogenomes have been determined, however, among these determined mitogenomes, only four are for the Zygaenoidea moths (Tang et al. 2014; Liu et al. 2016; Peng et al. 2017). In this study, we first reported the complete mitogenome of *Pidorus atratus* (Lepidoptera: Zygaenoidea: Zygaenidae). Adult individuals of *P. atratus* were collected from Mountain Huangshan, Anhui province, China in August 2017. After morphological identification, the specimens were kept in the laboratory at –80 °C until used for DNA extraction. Total genomic DNA was isolated and purified from a single leg using a DNA extraction kit (Sangon Biotech Co., Ltd, Shanghai, China) according to the manufacturer’s instructions. All the PCRs and PCR product sequencings were performed after Hao et al. (2013) and the resultant reads were assembled and annotated using the BioEdit 7.0 (Hall 1999) and MEGA 7.0 (Kumar et al. 2016).

The complete mitogenome of *P. atratus* is a circular molecule of 15,383 bp in size (GenBank accession MG882482), containing typical 37 genes for insects: 13 protein-coding genes (PCGs), 22 tRNAs, two ribosomal RNA genes (rRNAs), and one AT-rich region. The gene order and arrangement are consistent with all other lepidopterans (e.g. Yang et al. 2009; Sun et al. 2012; Hao et al. 2013; Wu et al. 2016). The A + T content of the whole mitogenome is 79.8%, which is generally in accordance with other four determined Zygaenoidea mitogenomes. All PCGs use typical ATN as start codon, except the cox1 which uses CGA as its start codon; 10 PCGs use the standard stop codon TAA, while cox1, cox2, and nad4 end with a single T. All tRNAs are folded into the cloverleaf secondary structures except *tRNA^Ser^* (AGN) which lacks the DHU arm. The *rrnL* (16S rRNA) and *rrnS* (12S rRNA) genes are 1366 bp and 544 bp in size, respectively. The putative AT-rich region is 658 bp in size, harbouring several structures characteristic of lepidopterans (Hao et al. 2013; Shi et al. 2015).

The phylogenetic tree of 44 lepidopteran species including the *P. atratus* of this study and other four determined Zygaenoidea species, which represent 11 lepidopteran superfamilies were reconstructed based on the concatenated 13 PCG nucleotide sequence data with the Bayesian inference (BI) method, using three Hepialidae species as the outgroups (see Figure 1 for details). The result showed the *P. atratus* formed a monophyletic group with other four Zygaenoidea species with strong support value, which contradicted with the result of previous study in that the two Zygaenoidea families, Zygaenidae and Limacodidae, constituted a paraphyletic group (Liu et al. 2016).

**Figure 1.**
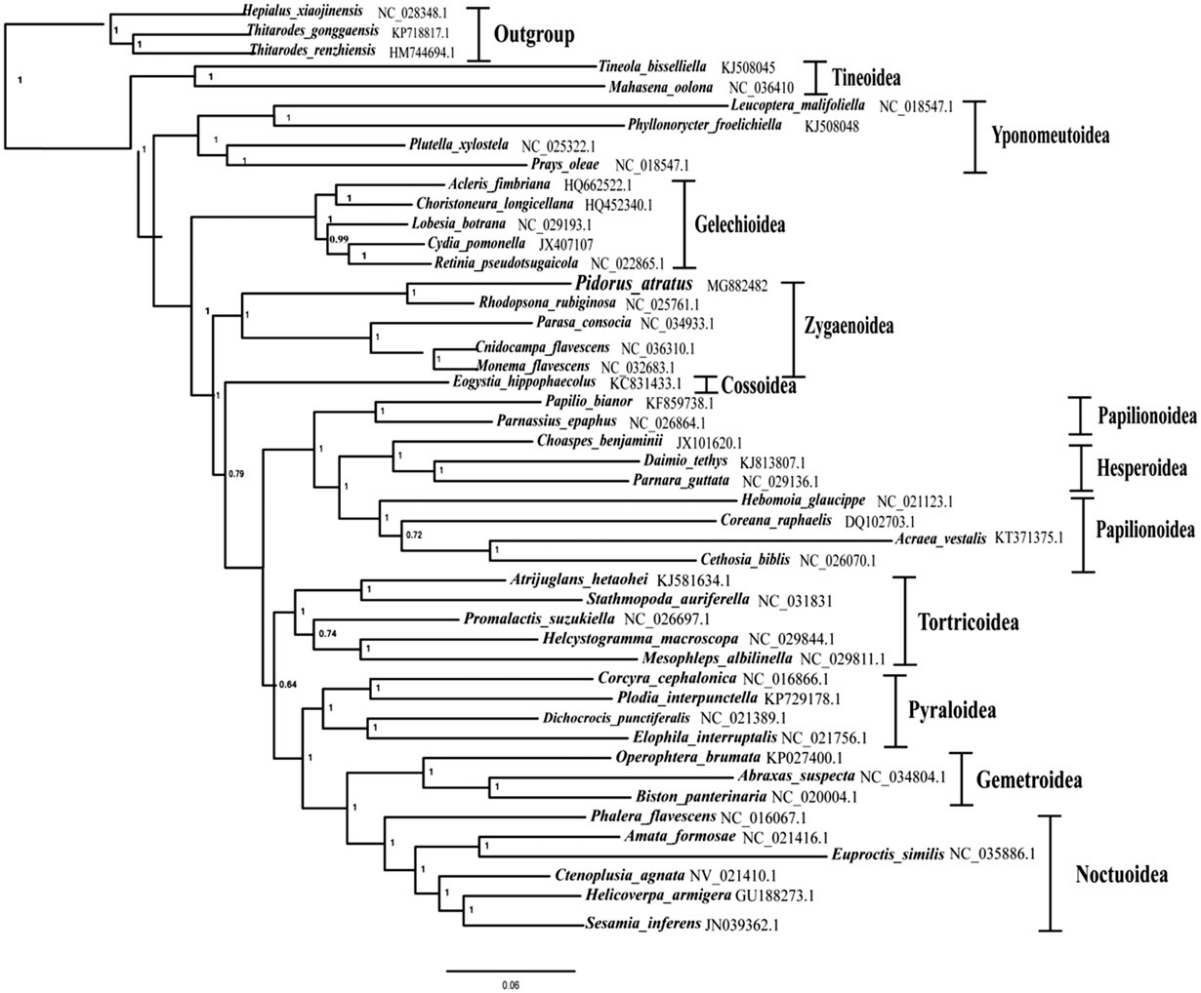
The Bayesian inference (BI) phylogenetic analysis tree of *P. atratus* and other lepidopterans. Phylogenetic reconstruction was done from a concatenated matrix of 13 protein-coding mitochondrial genes of the mitochondrial genome. The numbers beside the nodes are bootstrap values. Alphanumeric terms indicate the GenBank accession numbers.
